# Drépanocytose en République Démocratique du Congo: quels sont les obstacles à un traitement par hydroxyurée?

**DOI:** 10.11604/pamj.2021.38.41.18718

**Published:** 2021-01-15

**Authors:** Benoît Mbiya Mukinayi, Guelord Kalombo Cibeyibeyi, Ghislain Disashi Tumba, Béatrice Gulbis

**Affiliations:** 1 Faculté de Médecine, Université de Mbujimayi, Mbujimayi, République Démocratique du Congo,; 2 Hôpital Erasme, Université Libre de Bruxelles, Bruxelles, Belgique

**Keywords:** Prix, hydroxyurée, drépanocytose, pays en développement, Price, hydroxyurea, sickle cell disease, developing countries

## Abstract

**Introduction:**

l´hydroxyurée est le seul produit dont l´efficacité a été prouvée pour prévenir les complications de la drépanocytose et approuvé par la « Food and Drug Administration». Pour pouvoir offrir ce traitement aux patients atteints de drépanocytose, il doit être prescrit, disponible et à un prix abordable. L´objectif de cette étude est de déterminer la disponibilité et le coût de l´hydroxyurée sur le marché en République Démocratique du Congo (RDC) et comparer les deux aspects entre une ville reculée, à savoir Mbujimayi, et une grande ville, à savoir, Lubumbashi.

**Méthodes:**

il s´agit d´une étude transversale réalisée dans le cadre d´une enquête face-à-face menée du 1^er^ avril au 1^er^ septembre 2017 auprès de 188 pharmacies congolaises.

**Résultats:**

hydroxyurée était disponible dans 41/188 (22%) pharmacies participantes, mais plus fréquemment dans celles d´une grande ville que dans celles d´une ville reculée (34/96 contre 7/92). La plupart des patients ont reçu une ordonnance médicale (36/41; 88%). Le prix moyen de l´hydroxyurée était de 15 $ (10 $ à 35 $) pour une boîte de 25 gélules; ce prix est supérieur au pouvoir d´achat de la majorité des patients drépanocytaires. L´hydroxyurée reste encore un produit importé de l´Europe, des États-Unis ou d´Asie.

**Conclusion:**

l´hydroxyurée est l´un des principaux traitements permettant d´améliorer l´évolution de la maladie chez les patients drépanocytaires. Néanmoins, en République Démocratique du Congo, sa disponibilité pourrait être améliorée, en particulier dans une ville reculée, et son prix reste trop élevé pour être payé par la population locale.

## Abstract (en)

**Introduction:**

hydroxyurea is the unique medication that has been proven to prevent complications in patients with sickle cell disease and is approved by the Food and Drug Administration. This medication requires a prescription to be dispensed, it must be available and at an affordable price. The purpose of this study was to determine the availability and market price of hydroxyurea in the Democratic Republic of the Congo and to make a comparison between these two aspects in a small city, such as Mbujimayi, and in a big city, such as Lubumbashi.

**Methods:**

we conducted a cross-sectional study in the context of a face-to-face survey involving 188 Congolese pharmacies from 1^st^ April to 1^st^ September 2017.

**Results:**

hydroxyurea was available at 41/188 (22%) participating pharmacies, but more frequently at those of a big city than at those of a small city (34/96 versus 7/92). Most patients got a prescription (36/41; 88%). The average price of hydroxyurea was $15 (from $10 to $35 a blister packs of 25 capsules), which was higher than the purchasing power of the majority of sickle cell patients. Hydroxyurea is still an imported product from Europe, the United States or Asia.

**Conclusions:**

hydroxyurea is one of the main treatments to slow down disease progression in sickle cell patients. Nevertheless, in the Democratic Republic of the Congo, its availability could be improved, in particular in small cities, and its price is still too high.

## Introduction

La drépanocytose est une des maladies fréquentes en RDC comme le prouvent les données épidémiologiques récentes. Elles ont montré que 2% des nouveau-nés sont homozygotes pour l´hémoglobine S et environ 40.000 naissances d´enfants drépanocytaires sont estimées chaque année [1,2]. Si ce chiffre est significatif au point de vue épidémiologique, la maladie reste peu connue avec pour conséquence une forte mortalité dans un pays à ressources limitées [3]. Cliniquement, la drépanocytose se manifeste par des épisodes d´anémies aiguës, des crises douloureuses appelées crises vaso occlusives et la susceptibilité aux infections [4].

Les enfants drépanocytaires qui présentent un tableau clinique sévère comme des crises vaso-occlusives répétées, un risque élevé de vasculopathie cérébrale, un épisode de syndrome thoracique aigu ou une anémie chronique sévère doivent bénéficier l´un des trois traitements actuellement disponibles: l´hydroxyurée, des transfusions sanguines chroniques ou une transplantation de cellules souches [5]. Néanmoins, en Afrique subsaharienne, les transfusions de globules rouges restent à risque de transmission d´agents infectieux et la transplantation de cellules souches n´est pas disponible [6]. L´hydroxyurée est utilisée depuis 1980 et approuvée par la Food and Drug Administration pour son indication dans la prise en charge de la drépanocytose. Cette molécule est aujourd´hui largement utilisée depuis les résultats incontestables de l´essai randomisé chez les patients drépanocytaires adultes [7] et ceux obtenus chez les enfants drépanocytaires [8]. Ces études ont montré que l´hydroxyurée induit une augmentation de l´hémoglobine fœtale (HbF) qui interrompt la polymérisation de l´hémoglobine S à l´état désoxygéné. Comme le bénéfice clinique précède l´augmentation de l´HbF, d´autres mécanismes d´action ont été démontrés: une chute du nombre de leucocytes, une réduction de l´adhésion des GR à l´endothélium vasculaire et une augmentation de la production de NO [9]. Le bénéfice clinique a été démontré dans une étude belge randomisée sur 6 mois (hydroxyurée versus placebo; puis inversion des deux bras) entre autres par une chute du nombre d´hospitalisations et du nombre de jours d´hospitalisation [10].

Les indications de l´hydroxyurée en Afrique sont plus fréquentes du fait de l´inaccessibilité de la transfusion sanguine chronique et devrait faire l´objet d´un consensus [3]. L´hydroxyurée a pris une place significative en permettant de traiter et prévenir certaines complications majeures de la drépanocytose tout en ayant une toxicité faible. De plus, elle permet de limiter le recours aux transfusions sanguines pour lesquelles le risque infectieux et les problèmes d´approvisionnement subsistent en Afrique subsaharienne. Il est aussi démontré que même en-dehors de ces tableaux cliniques sévères, les patients traités par hydroxyurée ont une survie prolongée. Une proposition a été de traiter tous les patients et non pas uniquement ceux atteint d´une forme sévère de la maladie [11]. La posologie de l´hydroxyurée chez l´enfant est de 15-20 mg/kg/jour en une fois [12] alors que chez l´adulte la dose peut aller jusqu´à 35 mg/kg/jour en une prise [13]. Le coût annuel d´un traitement par hydroxyurée est estimé à 65 dollars chez un patient pesant 30 kg en moyenne.

Néanmoins cette molécule reste peu accessible en Afrique subsaharienne et son coût reste élevé [3,12]. Il y a très peu de données sur le prix réel de l´hydroxyurée dans les pays en développement où on trouve la majorité des patients drépanocytaires. Des études de terrain sont nécessaire pour se rendre compte de la disponibilité et du prix exact de l´hydroxyurée pour les patients drépanocytaires. Cette étude a pour objectif d´étudier la disponibilité et le prix de l´hydroxyurée dans deux villes de la RDC et faire une comparaison de cette disponibilité entre une ville reculée et une grande ville.

## Méthodes

Cette étude a été réalisée dans deux villes de la République Démocratique du Congo. La ville de Lubumbashi, Chef-lieu de la Province du Haut-Katanga au Sud, une de grandes villes coloniales de la RDC, et la ville de Mbujimayi, Chef-lieu de la Province du Kasaï oriental, une ville reculée au centre-Est de la RDC. C´est une étude transversale réalisée sur une période de 5 mois (1^er^ avril au 1^er^ septembre 2017). Le recrutement a été obtenu sur base de l´identification des pharmacies répondant aux critères d ´inclusion.

**Constitution de l´échantillon:** les interviews ont été menées par 4 étudiants en médecine (deux étudiants pour chaque ville) ayant bénéficié d´une formation sur la conduite de l´étude. Il a été procédé à un échantillonnage aléatoire au niveau chaque avenue. Pour chaque commune désignée ayant des activités commerciales dans chaque ville, les pharmacies à enquêter ont été sélectionnées après un travail d´identification des pharmacies. Celui-ci a été réalisé par les enquêteurs à l´issue de leur formation. C´était leur première visite de terrain. En effet, pendant 5 mois (cfr ci-haut) et à leurs rythmes, les enquêteurs formés, encadrés par des superviseurs, ont sillonné toutes les communes de la ville de Lubumbashi (Kampemba, Ruashi, Katuba, Kenya, Kamalondo et Lubumbashi) et les communes de la ville de Mbujimayi (Bipemba, Dibindi, Diulu, Kanshi et Muya). Les enquêteurs s´étaient reparti les communes à enquêtés, eux de Lubumbashi en 3 communes par chacun tandis que ceux de Mbujimayi, l´un a enquêté dans 3 communes et l´autre dans 2 communes. Au cours de ce travail de terrain, chaque enquêteur devait interviewer les responsables des pharmacies identifiées et remplir un questionnaire. Nous nous sommes arrêtés à 188 pharmacies (Mbujimayi 92/252 pharmacies et Lubumbashi 96/428 pharmacies) à la suite à une répétition des mêmes réponses. Chaque pharmacie interviewée était représentée par un responsable répondant qui était le gérant (58 %), le propriétaire (22%) ou le pharmacien (20%).

**Pharmacies enquêtées**: était incluse, toute pharmacie autorisée à fonctionner en RDC (autorisation d´ouverture du ministère de la santé, numéro d´ordre du pharmacien, numéro du registre de commerce et de crédit mobilier, numéro d´identification nationale) et ayant consenti à participer à l´étude. A été exclue, toute pharmacie ne remplissant pas les normes légales de fonctionnement, et n´ayant complété que partiellement le questionnaire. La taille de l´échantillon a été basée sur le nombre des pharmacies répondant. Cette étude a été approuvée par l´Université de Mbujimayi et a fait l´objet d´une lettre de recommandation. Un consentement libre et éclairé a été obtenu de la part des enquêtés. Les données récoltées étaient analysées d´une manière anonyme.

**Questionnaire**: le questionnaire a été établi par une équipe de la faculté de médecine de l´université de Mbujimayi et validé par une étude pilote sur 25 pharmacies. Les questions ont abordé les points suivants: les données d´identification de la pharmacie et des documents légaux, la connaissance de l´hydroxyurée et sa disponibilité physique dans la pharmacie, le prix de vente, le point de vue sur l´accessibilité du prix aux patients, lieux d´approvisionnement en hydroxyurée, le rythme de vente, l´appréciation du pouvoir d´achat des patients par rapport au prix, l´achat avec ordonnance médicale ou pas etc.

La disponibilité en hydroxyurée a été définie comme la présence physique de ce produit dans la pharmacie au moment de l´enquête. L´accessibilité au prix de l´hydroxyurée était évaluée selon l´appréciation du répondant. L´évaluation du savoir sur la drépanocytose, sur l´existence de l´hydroxyurée et de son indication dans la prise en charge de la drépanocytose était sur simple aveu du répondant. Seules des questions fermées ont été posées, soit dichotomiques (ex. disponibilité de l´hydroxyurée - oui/non) ou à choix multiples (ex. prix d´hydroxyurée).

**Analyse statistique**: l´analyse statistique des données collectées a été faite à l´aide du logiciel EPI InfoTM 7 (USA, CDC Atlanta, 2010) version 7.2. Les données ont, selon le cas, été représentées par: le médian ou la moyenne et leur intervalle de confiance à 95% pour les paramètres numériques; les fréquences absolues ou relatives pour les paramètres caractériels. Le test paramétrique d´Anova a été utilisé pour comparer les moyennes avec un intervalle de confiance à 95%. Les valeurs p < 0,05 étaient considérées comme statistiquement significatives.

## Résultats

Sur l´ensemble de questionnaires lancés dans les deux villes, Lubumbashi et Mbujimayi, nous avons eu un taux de réponse de 90% soit 225/250 pharmacies ont répondu à notre questionnaire. Trente-sept pharmacies ont été exclues par suite d´un remplissage incomplet du questionnaire. Un total de 188 interviews auprès d´un répondant parmi les pharmacies a été collecté parmi lesquels 96 (51%) pharmacies de la ville de Lubumbashi et 92 (49%) pharmacies de la ville de Mbujimayi. L´hydroxyurée était disponible dans 41/188 (22%) pharmacies pour l´ensemble de 2 villes soit 7 pharmacies à Mbujimayi et 34 pharmacies à Lubumbashi. Le prix médian de l´hydroxyurée est de 15$US (10-35$) pour une boite de 25 gélules. Le prix de l´hydroxyurée était supérieur à 15$US dans la majorité de pharmacies (73%). Le prix de vente de l´hydroxyurée était supérieur au pouvoir d´achat de la majorité de patients 30/41 (73%). La majorité d´enquêté savait l´existence de la drépanocytose (76%) et du médicament « hydroxyurée » (80%), cependant, seulement 53 (28%) de répondants savaient que l´hydroxyurée était indiqué dans la prise en charge de la drépanocytose (Tableau 1). La majorité de clients de l´hydroxyurée dans les pharmacies enquêtées se présentaient avec une ordonnance médicale (88%). Les pharmacies enquêtées importent l´hydroxyurée de l´étranger dans 100% des cas et l´origine d´importation est majoritairement l´Europe 35/41 (86%).

**Tableau 1:** évaluation des connaissances des répondants et caractéristiques liées au prix de vente de l´hydroxyurée

Connaissances sur la drépanocytose (N=188)	Nombre (pourcentage)
Existence de la maladie (anémie SS)	142(76)
Existence de l´hydroxyurée	150(80)
Connaissance d´une thérapie par hydroxyurée dans la drépanocytose	53(28)
Disponibilité de l´hydroxyurée dans la pharmacie	41(22)
**Caractéristiques liées au prix de vente de l´hydroxyurée (**N=41)	
**Prix de vente de l´hydroxyurée : médiane (min-max) : 15$(10–35$)/boites de 25 gélules**	
Inférieur ou égal à 15 $/boite de 25 gélules	11(27)
Supérieur ou à 15 $/boite de 25 gélules	30(73)
**Vente sur ordonnance médicale**	36(88)
**Prix de l´hydroxyurée par rapport au pouvoir d’achat des patients**	
Supérieur	30(73)
Inférieur	11(27)
**Importation de l´hydroxyurée**	41(100)
**Origine d´importation**	
Etats-Unis d´Amérique	5(12)
Europe	35(86)
Asie	1(2)
**Un prix de l´hydroxyurée > 10 $/boîte est-il accessible aux patients**	10(24)
**Avez-vous des patients qui ont une prise chronique d´hydroxyurée ?**	25(61)
**Avez-vous des ordonnances d´hydroxyurée provenant des hôpitaux**	15(37)
**Rythme de vente annuelle de stock d´hydroxyurée**	
Moins de 50% du stock	10(24)
50 à 75% du stock	5(12)
Plus de 75% du stock	26(63)

**Comparaison de la disponibilité de l´hydroxyurée entre une ville reculée (Mbujimayi) et une grande ville (Lubumbashi)**: l´hydroxyurée est plus disponible à Lubumbashi, une grande ville, 34% (35/96) qu´à Mbujimayi, une ville reculée, 8% (7/92) (p < 0,0001). Le reste des paramètres étudiés sont décrit dans le Tableau 1.

## Discussion

Dans notre série, l´hydroxyurée était disponible dans 41/188 pharmacies soit 22%. Nous n´avons pas trouvé dans la littérature d´autres études semblables, néanmoins on constate un engouement des chercheurs sur l´intensification d´un traitement par hydroxyurée chez les patients drépanocytaires en Afrique subsaharienne. Beaucoup d´études sont en cours de réalisation ou à peine réalisée en Afrique subsaharienne afin d´étudier l´efficacité de l´hydroxyurée dans les régions à forte endémicité du paludisme. C´est notamment l´étude NOHARM [14] réalisée en Ouganda. L´étude REACH est un partenariat entre des chercheurs américains et africains visant à fournir des données sur la sécurité, la faisabilité et les avantages de l´hydroxyurée pour les enfants drépanocytaires en Afrique [9]. L´étude SPIN Trial est un essai de faisabilité pour déterminer l´acceptabilité du traitement par hydroxyurée pour la prévention primaire de l´accident vasculo-cérébral chez les enfants drépanocytaires présentant des résultats anormaux au doppler transcrânien [15]. D´autres études sur la drépanocytose en Afrique subsaharienne suggèrent entre autres que l´hydroxyurée, devraient être accessible sans réserve, avec des stratégies thérapeutiques codifiées. Il est important que des groupes d´influences scientifiques et des associations de patients des pays du Nord et du Sud s´associent pour faire prendre conscience aux gouvernements de l´urgence de mettre au point ces stratégies de prise en charge des malades [3]. Nos résultats montrent que l´hydroxyurée est moins disponible dans les régions reculées comparativement aux grandes villes en RDC. La situation de la drépanocytose n´est pas vraiment documentée dans les villes reculées des pays en développement. Dans notre récente enquête réalisée chez 50 familles concernées par la drépanocytose à Mbujimayi, aucun patient drépanocytaire n´était traité par hydroxyurée [16]. Ceci pourrait expliquer en partie ce manque d´intérêt de la part des pharmacies pour vendre le médicament.

Nous avons trouvé que le prix médian de l´hydroxyurée est de 15$US (10 - 35$US) pour une boite de 25 gélules. Dans les pays occidentaux, l´hydroxyurée coûte relativement moins cher et son achat sur prescription médicale, est remboursé intégralement [17,18]. Au regard de la situation socio-économique de la RDC, ce prix parait excessif au pouvoir d´achat de la population congolaise quand on sait qu´un congolais moyen vit avec moins de 1 $US /jour [19,20]. Les familles des drépanocytaires présentent un bas niveau socio-économique [21] et 62% des familles concernées par la drépanocytose n´ont pas un revenu mensuel suffisant pour la prise en charge de leur(s) enfant(s) [16]. Ce prix élevé de l´hydroxyurée dans notre série pourrait être lié aux différents taxes et difficultés des facilités fiscales en RDC [20]. Cependant, il est à noter que l´instauration d´un traitement par hydroxyurée dans la prise en charge de la drépanocytose est fondée sur la gravité de la maladie, et absolument pas sur le niveau de revenu économique des parents. Il serait souhaitable de réfléchir à des recommandations consensuelles d´utilisation de l´hydroxyurée en Afrique sub-saharienne [3]. Wang *et al*. rapportent que l´utilisation du traitement à l'hydroxyurée est associée à des économies substantielles sur les coûts médicaux du fait de ses avantages rapportés sur un traitement à long terme [22].

La plupart des patients se présentaient avec une ordonnance médicale (88%), ceci suggère que les médecins prescrivent ce médicament. Cependant, des études montrent que parmi les barrières au traitement par hydroxyurée, il y a l´insuffisance de prescription de celle-ci. Cette insuffisance de prescription serait liée à l´insuffisance des connaissances des médecins, les effets secondaires du médicament, l´idée que le patient ne serait pas compliant, etc. [23]. Notre enquête a porté sur les pharmacies et non pas sur les prescripteurs. Ce versant pourrait être complété dans une p-étude ultérieure. Nos enquêtés connaissaient l´existence de la drépanocytose (76%). Si elle est bien connue dans la population générale, elle l´est de manière incomplète [24]. En effet si l´existence était observée pour 80% des enquêtés, seulement 28% (53/188) savaient que ce traitement était indiqué dans la prise en charge de la drépanocytose. Les pharmacies enquêtées importent l´hydroxyurée de l´étranger dans 100% des cas. L´origine d´importation est majoritairement l´Europe (86%) suivi des Etats-Unis d´Amérique (12%) et l´Asie (1%).

## Conclusion

Malgré la prévalence élevée de la drépanocytose en RDC, la disponibilité de l´hydroxyurée dans les pharmacies est insuffisante, ceci laisse entrevoir l´impact sur la qualité de la prise en charge des patients drépanocytaires. Cette insuffisance de la disponibilité de l´hydroxyurée est plus marquée dans les villes reculées. Le prix élévé de l´hydroxyurée rend encore difficile l´accessibilité au traitement par cette molécule. Pour une intensification de ce traitement chez les patients drépanocytaires dans ce pays, il faut une politique d´approvisionnement et/ou de production locale de l´hydroxyurée générique à un prix accessible aux patients.

### Etat des connaissances sur le sujet

- La drépanocytose est une des maladies héréditaires récessives les plus fréquentes à travers le monde et en particulier en Afrique subsaharienne. On estime que 7% de la population mondiale sont porteurs d´une anomalie de l´hémoglobine (anémie falciforme et la thalassémie) et chaque année, 300 000 enfants naissent avec la maladie dont les 2/3 en Afrique subsaharienne; il est rapporté que près de plus de 50% d´enfants atteints par la drépanocytose meurent avant leur cinquième anniversaire s´ils ne sont pas suivis médicalement;
- L´hydroxyurée, établie depuis 1980 et approuvée par la Food and Drug Administration. Cette molécule est aujourd´hui utilisée dans la prise en charge de la drépanocytose. Ce médicament est moins disponible en Afrique subsaharienne ou on trouve la majorité des patients drépanocytaires;
- Plusieurs études sont lancées presque simultanément sur la nécessité d´instauration ou d´intensification d´un traitement par hydroxyurée dans la prise en charge de la drépanocytose dans les pays d´Afrique subsaharienne où sévit le paludisme et d´autres pathologies pouvant avoir des implications sur les effets de ce médicament.

### Contribution de notre étude à la connaissance

- Contribuer à une meilleure compréhension des barrières de la prise en charge de la drépanocytose en Afrique subsaharienne, notamment plaider pour le prix de vente de l´hydroxyurée dans les pharmacies pour les patients drépanocytaires, les différences entre les grandes villes et les villes reculées de ces pays, et en vue d´aider les décideurs locaux à proposer des stratégies appropriées d´approvisionnement en l´hydroxyurée et de la prise en charge de cette pathologie;
- Encourager et faciliter la recherche en vue d´améliorer la qualité de vie des malades.

## Remerciements

Nous remercions chaleureusement toute l´équipe de la faculté de médecine et les étudiants de troisième bachelier de médecine de l´université de Mbujimayi pour leur contribution multiforme (amendement du protocole, formation des enquêteurs, collecte et encodage des données, vérification des questionnaires, etc.). Nos remerciements s´adressent également à toutes les pharmacies qui ont accepté de participer à cette étude.
